# A Role for NKG2D in NK Cell–Mediated Resistance to Poxvirus Disease

**DOI:** 10.1371/journal.ppat.0040030

**Published:** 2008-02-08

**Authors:** Min Fang, Lewis L Lanier, Luis J Sigal

**Affiliations:** 1 Program of Viral Pathogenesis, Division of Basic Sciences, Fox Chase Cancer Center, Philadelphia, Pennsylvania, United States of America; 2 Department of Microbiology and Immunology, University of California San Francisco, San Francisco, California, United States of America; 3 Cancer Research Institute, University of California San Francisco, San Francisco, California, United States of America; Saint Louis University, United States of America

## Abstract

Ectromelia virus (ECTV) is an orthopoxvirus (OPV) that causes mousepox, the murine equivalent of human smallpox. C57BL/6 (B6) mice are naturally resistant to mousepox due to the concerted action of innate and adaptive immune responses. Previous studies have shown that natural killer (NK) cells are a component of innate immunity that is essential for the B6 mice resistance to mousepox. However, the mechanism of NK cell–mediated resistance to OPV disease remains undefined. Here we show that B6 mice resistance to mousepox requires the direct cytolytic function of NK cells, as well as their ability to boost the T cell response. Furthermore, we show that the activating receptor NKG2D is required for optimal NK cell–mediated resistance to disease and lethality. Together, our results have important implication towards the understanding of natural resistance to pathogenic viral infections.

## Author Summary

Ectromelia virus (ECTV) causes mousepox, a murine disease that is the equivalent of human smallpox. ECTV normally penetrates through the periphery but rapidly spreads through the lymphatic system to vital organs. In mousepox-sensitive strains of mice, ECTV infection culminates with either rapid death or overt symptoms of mousepox due to very high loads that the virus reaches in vital organs, particularly the liver. However, some strains of mice such as C57BL/6 (B6) and 129 also become infected with ECTV but naturally resist mousepox by controlling the virus loads in vital organs and clearing the virus without clinical symptoms of disease. Natural killer (NK) cells are cells of the innate immune system previously shown to play an important role in natural resistance to mousepox. However, how NK cells protect from this disease is still unknown. In this paper we show that NK cells directly contribute to antiviral defenses by curbing virus dissemination to vital organs and also indirectly by augmenting the antiviral T cell response. We also demonstrate that optimal protection requires the activating NK cell receptor NKG2D which facilitates killing of ECTV-infected cells. Our work has important implications for the understanding of natural resistance to viral disease.

## Introduction

Ectromelia virus (ECTV), the causative agent of mousepox, is an orthopoxvirus (OPV) with host specificity for the mouse. It is genetically very similar to vaccinia virus (VACV), the human pathogen variola virus (the agent of smallpox), and monkeypox virus [1], which sporadically infects people in Africa and produced a recent outbreak in the midwestern United States [2,3]. Like smallpox, mousepox is a severe disease with high mortality and infectivity. Thus, mousepox constitutes an excellent model to study smallpox and exanthematous diseases in general [4].

Although all mouse strains can be infected with ECTV, the outcome of the infection following footpad inoculation varies. Some sensitive strains, such as DBA/2, A/J, and BALB/c, develop mousepox and suffer high mortality during the first 2 wk post-infection, whereas other strains, such as C57BL/6 (B6), clear the infection without visible symptoms of systemic disease [5]. The resistance of B6 mice to mousepox is not due to an inherent decreased ability of the virus to replicate in this strain, but is a result of the combined action of the innate and adaptive immune systems [6–8].

Natural killer (NK) cells are innate effector cells serving as a first line of defense against certain viral infections and tumors [9,10]. For example, NK cell–deficient individuals become sick or succumb to normally non-life-threatening infections with the herpes virus human cytomegalovirus (HCMV) or varicella zoster [11–13]. In addition, C57BL/6 mice, which are normally resistant to mouse cytomegalovirus (MCMV), become susceptible when NK cells are depleted [14]. Similarly, NK cells have been shown to be crucial for the resistance of B6 mice to mousepox [15–17].

The activation of NK cells is regulated by a balance of signals transduced by activating and inhibitory receptors [18,19]. It is thought that continuous signaling through inhibitory receptors maintain NK cells in a resting state, and the loss of inhibitory signals (i.e., due to downregulation of MHC class I on target cells) or the expression of ligands for activating receptors on target cells results in NK cell activation [19]. Previous studies have shown that *Rmp1*, a dominant gene important for the ability of B6 mice to resist mousepox, maps to the “NK complex,” a region of chromosome 6 that encodes a large number of NK cell–activating and inhibitory receptors [16,18]. Well-defined activating receptors encoded by polymorphic genes within the NK complex in B6 mice include NKR-P1C (NK1.1, the prototypic marker defining NK cells in B6 mice), the Ly49 family members Ly49H and Ly49D, NKG2D, CD94-NKG2C, and CD94-NKG2E. *Cmv1*, a gene responsible for the resistance of B6 mice to MCMV infection and also mapped to the NK complex, has been shown to be the activating receptor Ly49H that binds to the MCMV-encoded MHC-like protein m157 expressed on the surface of infected cells [20–23]. On the other hand, the positive identification of any activating NK cell receptor involved in resistance to mousepox is still lacking, even though it has been speculated that *Rmp1* might be the prototypic activating receptor NK1.1 [24].

In this study, we show that NK cells directly contribute to antiviral defenses by curbing virus dissemination to central organs and also indirectly by augmenting the antiviral T cell response. We also demonstrate that the activating receptor NKG2D is involved in the NK cell–mediated resistance to mousepox, whereas NK1.1 is not.

## Results

### Resistance to Mousepox Requires NK Cells, but Only during the First 4 d Post-Infection

**Figure 1:**
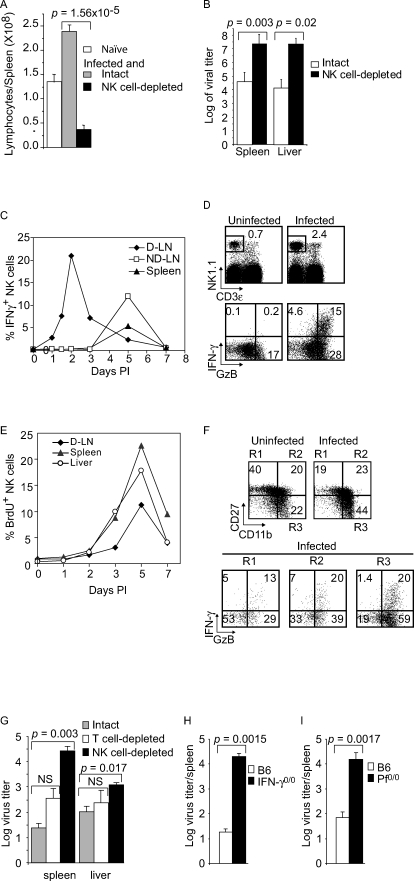
The Rapid Kinetics of the NK Cell Response Controls the Early Dissemination of ECTV to Central Organs by Using IFN-γ- and Perforin-Dependent Mechanisms Intact or NK cell–depleted (treated with anti-NK1.1 mAb PK136) B6 mice were infected with 3,000 pfu ECTV. (A) 7 d PI, the absolute numbers of live lymphocytes in the spleen were determined by trypan blue exclusion. Data correspond to the average ± SD of pooled spleens of three mice from five independent experiments. (B) 7 d PI, virus titers in spleen and liver were determined by plaque assay. Data correspond to the average ± SD of six individual mice from two independent experiments. (C) B6 mice were infected with ECTV in the footpad. The NK cell response, as determined by the percentage of NK cells expressing intracellular IFN-γ, at the indicated times PI was determined in the indicated organs. Data correspond to pools of three mice and are representative of three similar experiments. (D) Representative flow cytometric analysis of D-LN from mice infected for 2 d with ECTV and from control uninfected mice. Upper panel: Dot plots indicating the proportion of NK cells (NK1.1^+^, CD3ɛ^−^). Lower panel: GzB and IFN-γ production by gated NK cells (NK1.1^+^/CD3ɛ^−^ gate of the upper panels). Data correspond to pools of three mice and are representative of at least five experiments. >98% of cells stained with control Ig were in the lower left quadrant of the dot plots (not shown). (E) B6 mice were infected with ECTV, and NK cell (NK1.1^+^, CD3ɛ^−^) proliferation was determined at different times PI in the indicated organs by using a 3-h BrdU incorporation assay. Data are representative of three independent experiments. (F) Flow cytometric analysis of D-LN from mice infected for 2 d with ECTV and from control uninfected mice. Upper panels: Plots are gated on NK cells (NK1.1^+^, CD3ɛ^−^). Lower panels: Gated on R1, R2, and R3 populations from the upper-right (infected) plot. Data are representative of three independent experiments. (G) Intact B6 mice and B6 mice depleted of NK cells (treated with anti-NK1.1) or T cells (treated with anti-CD4 and anti-CD8) were infected with ECTV and virus titers in spleen and liver were determined on day 3 PI. Data are the average ± SD of six individual mice from two experiments. (H) Wild-type and IFN-γ-deficient B6 mice were infected with ECTV in the footpad, and virus titers were determined 3 d PI. Data are the average ± SD of three individual mice and is representative of two individual experiments. (I) Wild-type and Pf-deficient B6 mice were infected with ECTV in the footpad and virus titers were determined 3 d PI. Data are the average ± SD of three individual mice and are representative of two individual experiments.

B6 mice are normally resistant to mousepox ([5] and Table 1). To determine whether and when NK cells are required for resistance to mousepox, we depleted NK cells in B6 mice at different times following ECTV infection with either anti-asialo GM1 antiserum or anti-NK1.1 mAb PK136. Anti-asialo GM1 antiserum depletes NK cells and some activated T cells but not NKT cells, whereas PK136 depletes NK cells and NKT cells but not T cells. Thus, the use of both Abs allowed us to better distinguish the role of NK cells from that of other lymphocyte populations. As shown in Table 1, depletion of NK cells with either Ab on or before day 4 post-infection (PI) with 3,000 pfu ECTV resulted in very high mortality, while mock-depleted mice (normal rabbit sera for anti-asialo GM1 and mouse IgG2a for PK136) were still resistant (data not shown). On the other hand, resistance to mousepox was maintained when mice were depleted of NK cells on day 6 PI, indicating an important role for the physical presence of NK cells at the early, but not later, stages of infection. In additional experiments, we found that five out of five and two out of five PK136-depleted B6 mice succumbed when infected with 100 and 1 pfu ECTV, respectively. Identical results were obtained for BALB/c mice infected with the same virus stock. This shows that the susceptibility of B6 mice depleted of NK cells on the day of infection is similar to that of genetically susceptible BALB/c mice. In agreement with the increased mortality, NK cell depletion resulted in severe necrosis and splenic lymphopenia as compared to non-depleted (intact) B6 mice (Figure 1A). Furthermore, 7 d PI, NK cell–depleted mice had more than 10^3^-fold higher viral titers in both the spleen and liver than intact mice (Figure 1B), indicating a more severe systemic infection in the absence of NK cells. Thus, NK cells are required in the early phase of infection to control viral loads in central organs and to resist lethal mousepox.

**Table 1:**
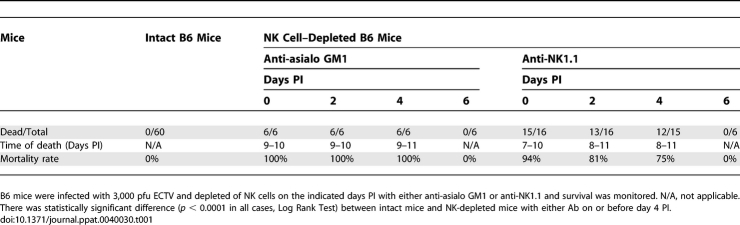
NK Cells Are Required for Resistance to Mousepox during Early ECTV Infection

### Early Recruitment of NK Cells to the Draining Lymph Nodes

We next evaluated the NK cell response at different times PI by flow cytometry. NK cell production of IFN-γ and granzyme B (GzB) in the draining lymph nodes (D-LN) was already induced 24 h PI, reaching the peak at 48 h PI when ∼20% of the NK cells produced IFN-γ and 43% produced GzB (Figure 1C and 1D), a time when T cell responses were not yet detected [25]. This was accompanied by a 2- to 4-fold increase in the proportion of NK cells in the D-LN (Figure 1D). NK cell responses in non-draining LN and spleen, however, did not peak until day 5 PI (Figure 1C). In addition, histopathological analysis of the infected footpads on day 2 PI did not show leukocyte infiltration or other signs of inflammation in either intact or NK cell–depleted B6 mice (data not shown). Thus, at very early stages of infection the NK cell response is restricted to the D-LN.

To distinguish whether the increase in NK cell number in D-LN resulted from recruitment and/or proliferation, we inoculated mice at different stages of infection with BrdU IP and sacrificed the mice 3 h later to determine BrdU incorporation by flow cytometric analysis. Very few NK cells incorporated BrdU in the D-LN during the first 3 d PI, suggesting increased migration to the D-LN at early times PI. In fact, on day 3 and 5 PI, the BrdU incorporation in NK cells in spleen and liver was higher than in the D-LN (Figure 1E). Thus, the increase of NK cells in the D-LN on day 2 PI is mostly due to recruitment rather than proliferation.

A recent report by Hayakawa and Smyth revealed that peripheral NK1.1^+^ NK cells can be divided into three subsets according to their expression of CD11b and CD27, which they designated R1 (CD27^+^, CD11b^−^), R2 (CD27^+^, CD11b^+^), and R3 (CD27^−^, CD11b^+^), that represent NK cells at distinct developmental stages [26,27]. R1 cells are immature, whereas R2 and R3 fractionate the mature cells into two populations. While adoptively transferred R2 cells can differentiate into R3 cells, R3 cells appear to be terminally differentiated. We, therefore, investigated whether ECTV infection altered the maturation phenotype of NK cells in the D-LN and found a substantial increase in NK cells with a mature (R3) phenotype on day 2 PI (Figure 1F, upper panels). Of interest, the majority of effectors (IFN-γ^+^ and/or GzB^+^) were within this mature NK cell population (Figure 1F, lower panels). Together, these data demonstrate that the increased NK cell number in the D-LN at early time points PI is mainly a result of recruitment. Moreover, the data show that the NK cells responding to ECTV infection are mature or mature very rapidly after infection.

### Control of Early Virus Dissemination by NK Cells Is Dependent on IFN-γ and Perforin

We hypothesized that the observed early recruitment to and activation of NK cells in the D-LN may contribute to the prevention of virus dissemination to central organs. Thus, we depleted NK cells in B6 mice with anti-NK1.1 mAb (PK136) and determined viral titers in spleen and liver on day 3 PI. To rule out a role for T cells at this stage we also depleted both CD4^+^ and CD8^+^ T cells by using a combination of anti-CD4 (GK1.5) and anti-CD8 (2.43) mAbs. Depletion of NK cells resulted in >10^3^-fold increase in viral loads in the spleen, while a much lower increase in virus titers was observed in mice depleted of T cells. The slight increase in virus titers in the T cell–depleted mice was not statistically significant (*p* = 0.09) and was not reproducible. We also observed a significant increase in virus titers on day 3 PI in the livers of NK cell–depleted mice, but not T cell–depleted mice (*p* = 0.07) (Figure 1G). Therefore, NK cells, and not T cells, are responsible for limiting the early dissemination of ECTV from the site of infection to central organs.

NK cells can control viral infections by producing antiviral cytokines, such as IFN-γ, or by perforin (Pf)-mediated killing of infected cells [28]. Others have shown that B6 mice deficient in either Pf or IFN-γ are susceptible to mousepox [29,30]. However, whether the early control of virus dissemination (day 3 PI) by NK cells requires IFN-γ and/or Pf-mediated killing is unknown. We, therefore, compared virus titers on day 3 PI in the spleens of wild-type (WT), IFN-γ-deficient, and Pf-deficient B6 mice. We found that both IFN-γ-(Figure 1H) and Pf-deficient mice (Figure 1I) had significantly elevated virus titers as compared to B6 mice. Thus, the early control of virus dissemination by NK cells requires both the antiviral effects of IFN-γ and Pf-mediated cytotoxicity.

### Reduced T Cell Response in the Absence of NK Cells

**Figure 2:**
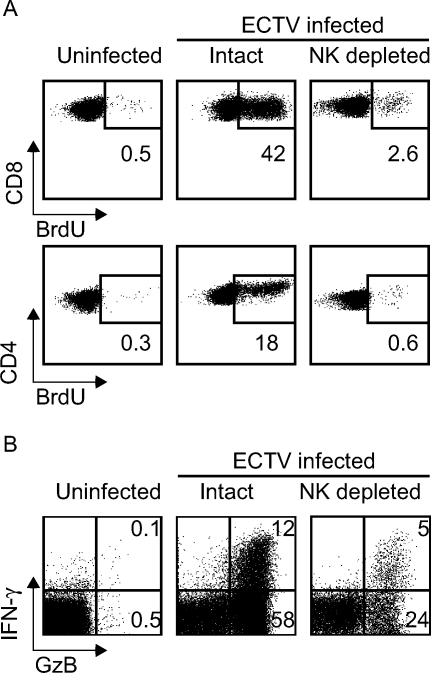
Reduced T Cell Responses in the Absence of NK Cells (A) Intact or NK cell–depleted B6 mice were infected with 3,000 pfu ECTV. T cell proliferation on day 5 PI was determined in the D-LN by using a 3-h BrdU incorporation assay. Upper panel: gated on CD8^+^ T cells; lower panel: gated on CD4^+^ T cells. (B) Intact or NK cell–depleted B6 mice were infected with ECTV. Production of IFN-γ and GzB by CD8^+^ T cells in spleen on day 7 PI was determined. Plots are gated on CD8^+^ T cells. Data are representative of three independent experiments.

Our previous studies and those of others showed that an optimal CD8^+^ T cell response is required for resistance to mousepox [8,25,31]. The data above indicate that NK cells have a direct effect in preventing mousepox by curbing virus dissemination during early infection. However, NK depletion at early stages resulted in an increase in spleen and liver viral titers, splenic lymphopenia, liver pathology, and death on day 7 PI (Figure 1 and Table 1), a time when the presence of NK cells was no longer necessary (Table 1) and when the T cell response should have already peaked [25]. This suggested that the absence of NK cells may have an impact on the establishment of the adaptive anti-ECTV T cell response. Thus, we compared in vivo T cell proliferation in intact mice and mice depleted of NK cells on day 0 PI. We found that on day 5 PI (when the T cell response normally peaks in D-LN), only a few CD8^+^ and CD4^+^ T cells incorporated BrdU in the D-LN of NK cell–depleted mice, while ∼40% of CD8^+^ T cells and 20% of CD4^+^ T cells incorporated BrdU in intact mice (Figure 2A). Mice depleted of NK cells on day 0 PI also had substantially reduced T cell proliferation in spleen and liver on day 7 PI (the time when the T cell response normally peaks in the spleen, data not shown). We also determined the CD8^+^ T cell response by intracellular staining for the effector molecules, GzB and IFN-γ. On 7 d PI, there was a strong T cell response in spleen with ∼70% of CD8^+^ T cells producing GzB and more than 10% of CD8^+^ T cells producing IFN-γ in intact B6 mice (Figure 2B). However, in mice depleted of NK cells on day 0 PI, the CD8^+^ T cell response was greatly reduced, with only ∼30% of CD8^+^ T cells producing GzB and ∼5% CD8^+^ T cells producing IFN-γ. Furthermore, because infected NK cell–depleted mice had severely lymphopenic spleens (Figure 1A), they also had a 16-fold reduction in the absolute number of effector CD8^+^ T cells as compared with infected intact mice (data not shown). These results indicate that the adaptive T cell response was substantially reduced in the absence of NK cells.

### Resistance to Mousepox Involves the Activating Receptor NKG2D but Not NK1.1

To gain insight into the mechanism whereby NK cells become activated during ECTV infection, we explored whether various known NK cell–activating receptors are involved in resistance to mousepox. We first focused on NK1.1 and on the activating receptors of the Ly49 family. We focused on these receptors because NK1.1 is not encoded in mousepox-susceptible DBA/2 and BALB/c mice. Furthermore, it has been speculated that NK1.1 might be *Rmp1* [24]. In addition, there is good precedent for the involvement of Ly49H in NK cell–mediated antiviral responses, more specifically to MCMV [20–22]. Ly49D and Ly49H are the only activating receptors of the Ly49 family in B6 mice. Because B6 mice selectively deficient in NK1.1, Ly49H, or Ly49D are not available, we first used 129/Sve mice, which do not express any of these receptors [22]. Similar to B6 mice, 129/Sve mice were naturally resistant to mousepox but became sensitive when depleted of NK cells with anti-asialo GM1 Ab (PK136 could not be used because 129/Sve mice do not express NK1.1) (Table 2). This demonstrates that NK cells are also required for 129/Sve mice resistance to mousepox but that NK1.1, Ly49D, or Ly49H are not essential for resistance because 129/Sve do not possess these receptors.

**Table 2:**
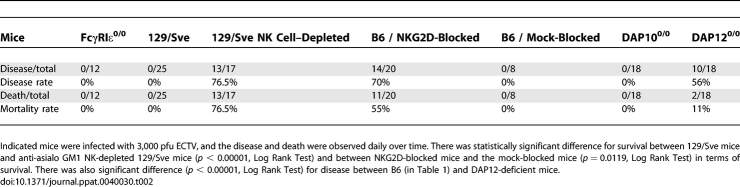
NKG2D Is Involved in NK Cell–Mediated Resistance to Mousepox

The results above suggested, but did not formally prove that Ly49H, Ly49D, and/or NK1.1 are not required for the resistance of B6 mice to mousepox, because it remained possible that Ly49H, Ly49D, and/or NK1.1 play a role in B6 resistance but that other activating receptors substitute their function in 129/Sve mice. Although it is not possible to definitively rule out the participation Ly49H or Ly49D in B6 resistance with currently available reagents, we took advantage of FcɛRIγ-deficient B6 mice [32] to rule out a role for NK1.1. It is known that NK1.1 signaling requires association with the ITAM-containing FcɛRIγ adapter [33]. FcɛRIγ-deficient B6 mice, however, were completely resistant to mousepox (Table 2), demonstrating that NK1.1 is not essential for the resistance of B6 mice to mousepox. In addition, because Abs are required for long-term resistance to mousepox [8,31] and FcɛRIγ is required for Ab-dependent cellular cytotoxicity (ADCC) [32], these data also demonstrate that ADCC is not involved in Ab-dependent resistance to mousepox.

We addressed whether the activating receptor NKG2D might be involved in mousepox resistance. NKG2D is expressed by NK cells and some T cells, including γδ-TcR^+^ T cells and activated CD8^+^ T cells. Although not polymorphic, this activating receptor is expressed at somewhat lower levels on activated NK cells of mousepox-susceptible NOD and BALB/c mice than in B6 mice ([34] and unpublished data). Because NKG2D-deficient mice are not available, we took advantage of the anti-NKG2D mAb CX5 that, in vivo, blocks binding of NKG2D to its ligands and causes its internalization without depleting NKG2D-bearing cells [35–37]. We inoculated B6 mice with CX5 mAb or control rat IgG 1 d before and 2 d PI. As shown in Table 2, CX5 treatment resulted in 50% mortality suggesting that NKG2D is involved in the NK cell–mediated resistance to mousepox.

In mice, NKG2D signals through either DAP10 or DAP12 adapter proteins, whereas Ly49H and D signal through DAP12 [38–40]; therefore, we determined mousepox susceptibility in either DAP12- or DAP10-deficient mice on a B6 mousepox-resistant background. We found that more than 50% of DAP12-deficient mice developed the typical skin rash, but only 10% died following ECTV infection, whereas all DAP10-deficient mice survived without showing ostensible symptoms of mousepox (Table 2).

**Figure 3:**
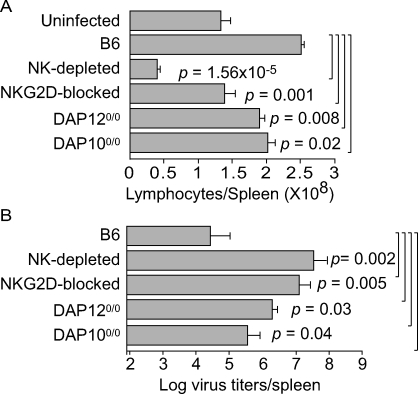
NKG2D Uses DAP10 or DAP12 Adapters to Help Resist Mousepox (A) The indicated mice were infected with 3,000 pfu ECTV and the absolute number of lymphocytes in their spleens was determined on day 7 PI by trypan blue exclusion. An uninfected control is also shown. Data are the average ± SD of three pooled mice per group from at least three individual experiments. (B) Virus titers in spleen of the indicated mice on day 7 PI. Data are the average ± SD of three pooled mice per group from at least three individual experiments.

To get further insights into the role of NKG2D and its adapters in resistance to mousepox, we determined cell numbers and virus titers 7 d PI in NKG2D-blocked as well as in DAP12- and DAP10-deficient mice. Results showed that NKG2D blockade and DAP12- or DAP10-deficiency resulted in significantly decreased splenic lymphocytosis as compared with wild type, untreated B6 mice (Figure 3A). More important, the viral loads in NKG2D-blocked mice were almost 10^3^ times higher than in unblocked mice, whereas those of DAP12- and DAP10-deficient mice were also significantly elevated, but to a lesser degree (10^2^ and 10 times higher, respectively) (Figure 3B). Thus, the degree of splenic lymphocytosis and viral loads on day 7 PI was consistent with the lethality and symptoms of mousepox that we observed under the different conditions. Together, these results show that NKG2D is involved in resistance to mousepox. The data also suggest that for this process NKG2D preferentially, but not exclusively, uses DAP12 as the signaling adapter. Most likely, the absence of one adapter is partially compensated by the presence of the other. The data further suggest that Ly49H and Ly49D are not essential for B6 resistance to mousepox because DAP12-deficient mice were only mildly susceptible to mousepox infection. This contrasts with prior studies demonstrating that DAP12-deficient mice on a B6 MCMV-resistant background are exquisitely sensitive to MCMV infection [41].

### NK Cells Require NKG2D to Control Early Virus Dissemination and to Mediate Optimal Cytotoxicity

**Figure 4:**
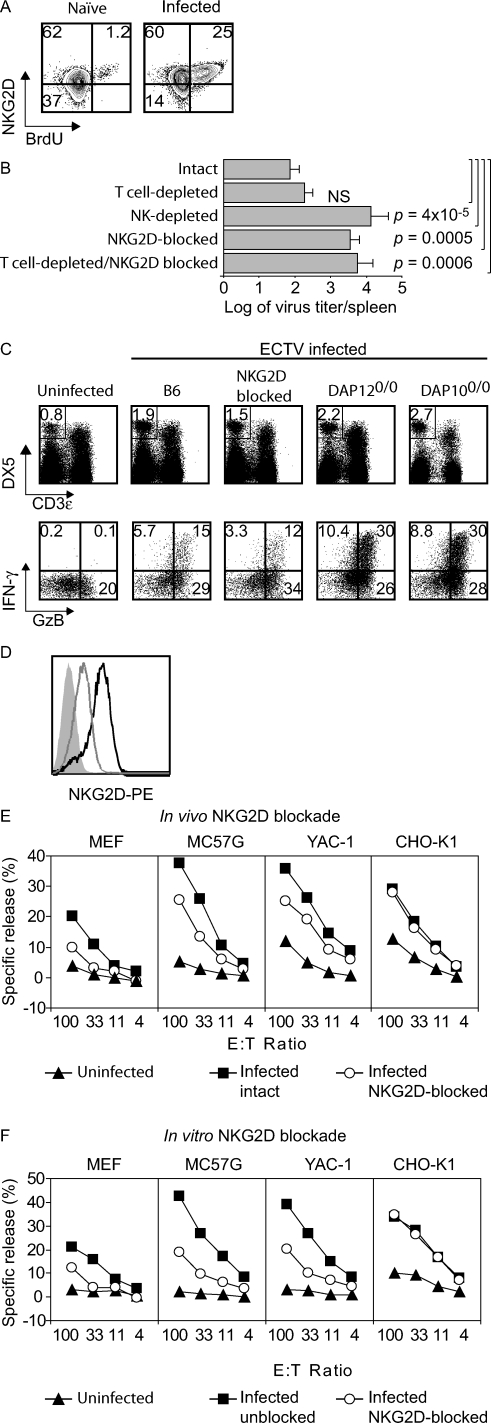
NK Cells Require NKG2D to Control Early Virus Dissemination and for Optimal Cytotoxicity but Not Activation (A) B6 mice were infected with 3,000 pfu ECTV. On day 5 PI, mice were pulsed with BrdU for 3 h and their spleens analyzed by flow cytometry. Plots are gated on CD3ɛ^−^ NK1.1^+^ cells. Data correspond to pools of three mice from three individual experiments. (B) Increased viral titers of infected mice with NKG2D blockade in vivo. Intact B6 mice, B6 mice with NKG2D blockade, B6 mice depleted of NK cells, B6 mice depleted of T cells (treated with anti-CD4 + anti-CD8 mAbs), or depleted of T cells and with NKG2D blockade were infected with 3,000 pfu ECTV and the viral titers in spleen were determined on day 3 PI. Data are the average ± SD of three individual mice and are representative of two similar experiments. (C) The indicated mice were infected with 3,000 pfu ECTV, and the NK responses in the D-LN were determined at 2 d PI. Upper panel: Dot plot showing the proportion of NK cells (DX5^+^, CD3e^−^) in the D-LN of infected and control uninfected mice. Lower panel: GzB and IFN-γ production by NK cells. Cells correspond to the DX5^+^, CD3ɛ^−^ gate of the upper panels. Data correspond to pools of three mice and are representative of three similar experiments. (D) NK cells were purified from spleens (5 d PI) of ECTV-infected intact or NKG2D-blocked mice and stained as indicated. Filled histogram: isotype-matched control Ig; gray line: NKG2D-blocked mice; black line: intact mice. (E) NK cells were purified from spleens of intact or NKG2D-blocked mice as indicated and were used as effectors in a 4-h ^51^Cr release assay against the indicated targets. Data correspond to pools of three mice and are representative of three experiments. (F) As in (D), but the NK cells were purified from untreated mice and a neutralizing anti-NKG2D mAb was added to the in vitro cytotoxicity assays, as indicated.

NKG2D is expressed by NK cells [42] and its expression is further increased during MCMV infection (J. Sun and L. L. Lanier, unpublished data). We, therefore, determined whether ECTV infection also increased NKG2D expression on NK cells. As shown in Figure 4A, NK cells increased NKG2D expression at the cell surface, and the proliferating NK cells after ECTV infection were NKG2D^high^.

The data above showed that NK cells are the main population-controlling early virus dissemination to visceral organs, and that absence of NKG2D signaling results in increased susceptibility to mousepox. However, NKG2D is not only expressed by NK cells but also by some T cells. We hypothesized that if NKG2D has a role in NK cell–mediated resistance to mousepox, NKG2D blockade should result in increased early virus dissemination to organs independent of T cells. To test this hypothesis, we measured early viral titers in the spleens (day 3 PI) of mice treated with the anti-NKG2D mAb CX5. To rule out a role of NKG2D on T cells at this time, we also included groups of mice that were T cell–depleted or NKG2D-blocked and T cell–depleted. As shown in Figure 4B, NKG2D-blocked and T cell–depleted + NKG2D-blocked mice had virus titers that were comparable to those of NK cell–depleted mice and that were significantly higher than those of mice that were only T cell–depleted. Together, these data indicate that NKG2D is involved in the NK cell–mediated resistant to mousepox.

We next tested whether NKG2D blockade and DAP10- or DAP12-deficiency affected the recruitment of NK cells to D-LN (Figure 4C, upper panels) or their ability to produce IFN-γ and GzB (Figure 4C, lower panels). However, we found that none of these parameters were decreased. In fact, in both DAP10- and DAP12-deficient mice more NK cells were recruited to the D-LN and a larger proportion produced IFN-γ and GzB as compared with wild-type B6 mice. This indicates that NKG2D is required for effective NK cell–mediated control of ECTV, but is not required for their activation.

Because NK cell recruitment and activation in the D-LN was not diminished by NKG2D blockade or DAP12 and DAP10 deficiency (Figure 4C), we hypothesized that NKG2D may be required for optimal NK cell–mediated cytotoxicity. Thus, we examined whether NK cells from NKG2D-blocked mice were defective in their ability to kill various targets. For this purpose, mice were infected with ECTV and treated or not with anti-NKG2D Ab. Five days later, NK cells were purified from their spleens and immediately analyzed by flow cytometry to confirm NKG2D receptor blockade or downregulation in the CX5-treated mice (Figure 4D) and were used as effectors in 4-h ^51^Cr release assays. Because we were unable to show specific killing of ECTV-infected cells in vitro, we tested the purified NK cells against a variety of uninfected targets that constitutively express NKG2D ligands at their cell surface (MC57G, MEF, and YAC-1) or, as a control, CHO-K1 whose killing is dependent on Ly49D [43,44], but not NKG2D. We found that the NK cells from mice infected with ECTV (whether treated with anti-NKG2D Ab or not) killed all targets much more effectively than those from uninfected mice. This enhanced killing did not require the targets to be infected. However, the NK cells from mice treated with CX5 were less effective killers of NKG2D ligand-bearing target cells as compared to those from untreated mice, except against CHO-K1 cells for which the NK cell killing is dependent on Ly49D and not on NKG2D (Figure 4E). Furthermore, purified NK cells from ECTV-infected intact mice also demonstrated reduced, but not absent cytotoxicity when anti-NKG2D mAb was added to the assay (Figure 4F). Thus, ECTV infection enhances spontaneous NK cell–mediated cytotoxicity, which is partially reduced by NKG2D blockade. Together, these data suggest that NKG2D signaling is not required for the recruitment and activation of NK cells during ECTV infection, but contributes to their optimal ability to kill targets expressing NKG2D ligands. Because anti-NKG2D did not reduce cytotoxicity to the same levels as those in uninfected mice, the data also indicate that activating receptors other than NKG2D are involved in the cytotoxicity induced by ECTV infection.

### ECTV Infection Upregulates NKG2D Ligands In Vitro and In Vivo

**Figure 5:**
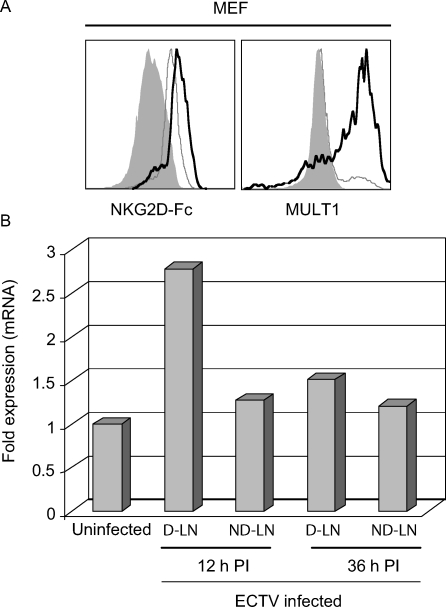
ECTV Infection Induces Increased Expression of NKG2D Ligands In Vitro and In Vivo (A) MEFs were infected with 0.5 pfu ECTV 189898-p7.5-EGFP for 18 h. Cells were analyzed for staining with the indicated reagents after gating for EGFP^−^ cells (uninfected) and EGFP^+^ cells (infected). Data correspond to one typical experiment from three similar experiments. Shaded area, infected cells stained with isotype-matched control Ig or secondary Ab alone; black line, infected cells stained with the indicated reagent; gray line, non-infected cells stained with the indicated reagent. (B) qRT-PCR was performed as described. Data were normalized to the amount of β-actin mRNA. Data are representative of two similar experiments.

NKG2D-mediated killing requires the recognition of ligands on the surface of target cells. The ligands of NKG2D are host cell–encoded MHC class I–like proteins that are expressed by tumors and stressed cells and also following infection of cells with some viruses. Identified cellular ligands for NKG2D in mice include H60, MULT1, and Rae-1 [45–49]. To determine if ECTV infection induces the upregulation of NKG2D ligands, we infected mouse embryo fibroblasts (MEFs) with 0.5 pfu/cell ECTV expressing enhanced green fluorescence protein (EGFP), and the expression of NKG2D ligands on infected and uninfected cells was determined by flow cytometry. Consistent with the ability of ECTV-activated NK cells to spontaneously kill them, MEFs constitutively express NKG2D ligands as revealed by staining with mouse NKG2D-Fc fusion protein [50] (Figure 5A). However, infection with ECTV increased NKG2D-Fc staining and resulted in clear upregulation of MULT1 (Figure 5A) but not Rae-1 (data not shown). We also observed increased staining with NKG2D-Fc and anti-pan Rae-1 and anti-MULT1 mAbs in the fibrosarcoma cell line MC57G, and an increase in staining with NKG2D-Fc and anti-pan Rae-1 mAb in peritoneal lymphocytes (data not shown). Moreover, using quantitative RT-PCR, we detected a 2.8-fold increase of Rae-1 transcripts in the D-LN of ECTV-infected mice at 12 h PI, as compared with uninfected controls (Figure 5C). Thus, these results show that ECTV infection can induce the expression of NKG2D ligands, suggesting that binding of NKG2D with these ligands might result in improved NK cell killing of infected cells in vivo and better control of ECTV dissemination.

## Discussion

In this study, we confirmed previous reports demonstrating that NK cells are required for natural resistance to mousepox [15–17]. More importantly, we define several of the mechanisms whereby NK cells afford this resistance.

To determine whether and when NK cells are required for resistance to mousepox we depleted mice of NK cells with either anti-asialo GM1 or anti-NK1.1 Abs at different times PI. Although separately these two approaches have caveats, together they provide conclusive evidence that the presence of NK cells during the first 4 d PI, but not beyond day 5 PI, is essential for resistance to mousepox. First, even though anti-NK1.1 Ab depletes NKT cells, the loss of mousepox resistance upon Ab treatment cannot be due to the elimination of NKT cells because anti-asialo GM1 Ab does not eliminate this population [51,52]. Furthermore, Parker et al*.* recently demonstrated that NKT cells are dispensable for resistance to mousepox because ECTV infection of mice deficient in NKT cells (i.e., CD1d- and Vα14Jα281 TCR-deficient mice) did not result in symptoms of mousepox or an increase in virus titers when compared to wild-type B6 mice [17]. Second, although anti-asialo GM1 can eliminate some activated T cells [53], the loss of resistance cannot be attributed to T cell depletion because asialo GM1 is not expressed on virus-specific T cells within the first few days after viral infection. Moreover, we show that anti-NK1.1 and -asialo GM1 Abs do not affect resistance when given on day 6 PI (the peak of the T cell response). Furthermore, treatment with anti-NK1.1, but not depletion of T cells one day before infection, resulted in significantly increased virus titers on day 3 PI. In addition, treatment with anti-asialo GM1 Ab accelerated ECTV lethality in RAG-1-deficient mice, which lack adaptive immunity (data not shown). γδ T cell–deficient mice are also resistant to mousepox [17], indicating that the possible depletion of some γδ T cells by anti-asialo GM1 Ab cannot account for the dramatic increase in ECTV lethality after treatment with this Ab. Although work in other laboratories has already shown that depletion of NK cells with either anti-NK1.1 or asialo-GM1 results in susceptibility to mousepox [6,15,17], depletion at different times PI has not been described previously. Sequential depletion of NK cells allowed us to establish that the presence of NK cells is required only during the very early stages of infection. This is consistent with the concept that NK cells serve as the first line of defense after infection, thereby providing sufficient time to mount a full-fledged T and B cell response. We also established, however, that the contribution of NK cells to mousepox resistance extends beyond the need for their physical presence to kill virus-infected cells early after infection because NK cell depletion before day 4 PI resulted in higher virus titers and death after this time. This is most likely due to the role of NK cells in allowing a potent antiviral T cell response to develop, as discussed below.

We also followed the kinetics of NK proliferation and activation in spleen, liver, and D-LN of ECTV-infected mice. Interestingly, although we detected some proliferation, we did not find activated NK cells in liver and spleen on day 3 PI. This was despite the fact that NK cell–mediated control of virus loads in spleen and liver already occurred at this time PI. In fact, the peak of NK cell activation in spleen and liver took place on day 5 PI. On the other hand, the proportion of total NK cells, as well as the proportion of activated NK cells in the D-LN, peaked as early as day 2 PI, and these parameters were still substantially elevated on day 3 PI, notwithstanding that the proliferation of NK cells at these times was not yet detectable in D-LN. In addition, we found that the increase in NK cell numbers in the D-LN on day 2 PI was mostly due to an increase in mature NK cells and that these mature NK cells were preferentially activated. Together, these data suggest that the prompt NK cell response in the D-LN is responsible for the early control of virus loads in central organs and that this response is mostly due to the recruitment and activation of mature NK cells rather than their expansion. To spread to central organs from the footpad, ECTV must pass through the D-LN [5,54–56]. Recently, we have shown that in mousepox-susceptible BALB/c mice, memory CD8^+^ T cells protect from mousepox, at least in part, by curbing the spread of ECTV from the D-LN to central organs and allowing for the establishment of a full-fledged adaptive response [54]. Our results here suggest that NK cells may use the same strategy, furthering a model where LNs are not only sites where lymphocytes are primed and proliferate but also the place where a major fight against virus spread takes place [54].

In addition to NK cells, strong adaptive T cell responses are essential for resistance to mousepox. Our experiments here show that NK cells have a direct role in controlling ECTV because they reduced virus loads on day 3 PI when the T cell response is still undetectable (data not shown and [25]). However, if the only role of NK cells were direct, one would expect mice depleted of NK cells to become sick, but recover once the adaptive immune system takes control. Yet, while the presence of NK cells on day 6 PI was not required for resistance to mousepox, their presence before day 6 PI was vital for controlling virus titers and preventing pathology and death after this time. This could be explained by the finding that depletion of NK cells on the day of infection resulted in a substantially reduced T cell response. There are three non-excluding possibilities that may explain this effect. First, NK cell production of cytokines such as IFN-γ may directly modulate the adaptive T cell response [28]. A caveat to this hypothesis is that NK cells produce as much or more IFN-γ in ECTV-infected mousepox-susceptible BALB/c and DBA2/J mice as in resistant B6 mice (unpublished data), but these susceptible mice fail to mount an effective T cell response to ECTV. Second, NK cells may indirectly affect the activation of T cells through their interaction with antigen-presenting cells such as macrophages and dendritic cells (DC). This hypothesis has the same caveats as the one above. Third, it is possible that in the absence of NK cells, the uncontrolled virus replication of a highly pathogenic virus overwhelms the T cell response. In support of this possibility, infection of mousepox-susceptible BALB/c mice with a highly attenuated strain of ECTV results in the induction of a strong T cell response (unpublished data), and depletion of NK cells does not affect the T cell response to the poorly pathogenic vaccinia virus (unpublished data) with which ECTV shares most of the dominant CD8^+^ T cell epitopes [57].

Previous work by Delano and Brownstein showed that *Rmp1*, a gene important for resistance to mousepox, maps to the NK complex in distal chromosome 6 [16]. Moreover, they proposed that *Rmp1* encoded NK1.1 [24]. However, we found that NK1.1 is not required for resistance to mousepox. In addition, our work suggests that the activating receptors Ly49D and Ly49H are likely not involved. On the other hand, using in vivo signaling blockade we found that NKG2D is involved in resistance to mousepox and that ECTV infection results in the upregulation of NKG2D ligands in vivo and in vitro. That NKG2D is involved in antiviral responses has precedents. For example, previous studies have shown that cytomegalovirus (CMV) induces the upregulation of NKG2D ligands and that mouse and human CMV encode immune evasion molecules that downregulate NKG2D ligands and are important for their pathogenesis [28]. However, a direct role for NKG2D in the response to poxviruses has not been described previously. Interestingly, Campbell et al*.* have recently shown that cowpox virus encodes a soluble competitive antagonist of NKG2D [58]. Although this gene has been lost in other OPVs, such as ECTV, VACV, VARV, and MPXV, these data support our finding that NKG2D is involved in resistance to some OPV infections. On the other hand, NKp30, NKp44, and NKp46, but not NKG2D, were found to be responsible for the recognition of VACV-infected cells by human NK cells [59]. Whether this reflects differences between NK cell recognition of different OPVs, differences between human versus mouse NK cells, or both, will require further investigation.

Because mouse NKG2D signals through either the adapter protein DAP12 or DAP10, we further tested the susceptibility of mice lacking one or the other adapter. Of interest, when considering virus titers and spleen cellularity, the susceptibility of these two strains of mice was intermediate between intact and NKG2D mAb–blocked B6 mice. This indicates that for resistance to mousepox, the two adapters likely have overlapping, but not completely redundant, effects. Still, DAP12 seems to be the preferred adapter because DAP12-deficient mice were more susceptible to mousepox than DAP10-deficient mice, although it is possible that other DAP12-associated receptors might contribute to resistance to mousepox. Our results predict that DAP10 + DAP12 double-deficient mice would be highly susceptible to mousepox. Unfortunately, these mice are not yet available.

NKG2D is expressed by most NK cells but also by activated CD8^+^ T cells. In fact, we have observed that in ECTV-infected B6 mice a large proportion of virus-specific CD8^+^ T cells express NKG2D beginning 7 d PI (unpublished data). Thus, the experiments reported here do not rule out the additional contribution of NKG2D in the T cell–mediated resistance to mousepox, a very interesting possibility that we are currently investigating. Nevertheless, our experiments clearly implicate NKG2D in the NK cell–mediated response to ECTV because in vivo NKG2D blockade resulted in enhanced virus loads in central organs before the onset of the T cell response and because in vivo NKG2D blockade reduced the cytotoxicity of ECTV-activated NK cells. Still, several lines of evidence indicate that NKG2D is not the only activating receptor involved in the anti-ECTV NK cell–mediated response and that its most likely role is as a co-stimulator that facilitates cytotoxicity rather than being required for the initial NK cell activation: (1) NK cell depletion was much more effective than NKG2D blockade at rendering B6 mice susceptible to mousepox; (2) NKG2D-blockade did not affect NK cell proliferation (data not shown), IFN-γ and GzB production by NK cells, or recruitment of NK cells into the D-LN of ECTV-infected mice; (3) In vivo and in vitro NKG2D blockade significantly decreased, but did not abrogate, the cytotoxicity of ECTV-activated NK cells. Ongoing studies in our laboratory are aimed at identifying other activating receptor(s) and signaling pathway(s) required for NK cell–mediated resistance to mousepox.

In summary, our work demonstrates that NK cells contribute to the natural resistance of B6 mice to mousepox by using direct effector functions (most likely the killing of infected cells) to curb virus dissemination and by supporting a strong adaptive T cell response. Moreover, our data suggest that the activating receptor NKG2D, but not NK1.1 or Ly49 family members, has a role in this NK cell–mediated resistance to mousepox by promoting optimal NK cell–mediated killing. Thus, our data provide substantial insights into the mechanisms of natural resistance to ECTV and possibly other OPV infections. Together, our work furthers our understanding of host-pathogen interactions and the mechanisms whereby NK cells protect from viral disease.

## Materials and Methods

### Cells.

YAC-1 and CHO-K1 cell lines were obtained from Dr. Kerry Campbell (Fox Chase Cancer Center, Philadelphia, Pennsylvania), and A9, MC57G, and BSC-1 cells were obtained from the ATCC. MEF cells were made from day 11 to 13 embryos from B6 mice. As standard tissue culture media, we used RPMI-10 that consisted of RPMI-1640 medium (Invitrogen) supplemented with 10% fetal calf serum (Sigma), 100 IU/ml penicillin and 100 μg/ml streptomycin (Invitrogen), 10 mM Hepes buffer (Invitrogen), and 0.05 mM 2-mercaptoethanol (Sigma). MEF were grown in DMEM medium containing 15% fetal calf serum. When indicated, RPMI 2.5 (as above but with 2.5% FCS) was used. When required, 10 U/ml interleukin 2 (IL2) was added to RPMI 10 (RPMI 10-IL2). All cells were grown at 37 °C and 5% CO_2_.

### Viruses, mice, and infections.

The production of ECTV stocks for infection of mice and the determination of titers in stocks and organs were done as described previously [8]. To generate ECTV 189898-p7.5-EGFP, we adapted the method described by Johnston and McFadden [60]. Briefly, a construct containing the ECTV Moscow fragment 189543–189897, the VACV early/late promoter p7.5, the sequence of EGFP, and the ECTV Moscow fragment 189950–190297, in that order, was made by recombinant PCR and cloned into plasmid Bluescript II SK+ to generate the targeting vector pBS-EVM189898-p7.5-EGFP. This targeting vector was used to transfect mouse A9 cells using Lipofectamine 2000 as per manufacturer's instructions (Invitrogen). The transfected cells were infected with wild-type ECTV (Moscow strain, 0.3 pfu/cell) in 6-well plates. 2 d later, transfected/infected A9 cells were harvested using a rubber policeman, frozen and thawed, and different dilutions of cell lysates were used to infect BSC-1 cells in 6-well plates. 2 h after infection, the cells were overlaid with media containing 0.5% agarose. 4 d later, green-fluorescent plaques were picked with a pipette tip and used to infect a new set of cells. The purification procedure was iterated five times until all plaques were fluorescent. The resulting virus, ECTV 189898-p7.5-EGFP, carries EGFP in a non-coding region and is as pathogenic as wild-type ECTV Moscow (not shown). For preparation of ECTV stock for infection of different cell lines, A9 cells were infected with 0.2 pfu ECTV/cell, and incubated at 37 °C, 5% CO_2_. After 5 d, the cells were collected, frozen and thawed three times, and then sonicated in a water-bath sonicator. The solid material was pelleted by centrifugation, and the supernatant was stored in aliquots at −80 °C. The DAP10-deficient mice [61,62] (generously provided by Dr. Joe Phillips) and DAP12-deficient mice [63] on the C57BL/6 background were bred at UCSF. All the other mice were bred at the Fox Chase Cancer Center Laboratory Animal Facility in specific pathogen-free rooms or were purchased from Jackson Laboratories. IFN-γ-deficient C57BL/6 mice were generously provided by Dr. Glenn Rall. For infections, sex-matched animals 8–12 wk old were transferred to a biosafety level 3 room. For ECTV infection, anesthetized mice were infected in the left footpad with 25 μl PBS containing 3 × 10^3^ pfu ECTV. Following infections, mice were observed daily for signs of disease (lethargy, ruffled hair, weight loss, skin rash, and eye secretions) and imminent death (unresponsiveness to touch and lack of voluntary movements). Moribund mice were euthanized by halothane inhalation. All of the experimental protocols involving animals were approved by the Fox Chase Cancer Center Institutional Animal Care and Use Committee.

For ECTV infection of cells, 3–5 × 10^5^ cells were plated in 6-well plates and cultured overnight to allow cells to adhere. The cells were then infected with 0.5 pfu ECTV/cell for 18 h, collected, washed, stained, and analyzed for surface expression of various markers. For ECTV infection of peritoneal cells, the mice were euthanized by halothane inhalation and injected i.p. with PBS, the abdomen massaged gently, and the peritoneal cells were collected by aspiration and washed.

### In vivo depletion of NK cells or T cells and NKG2D blockade.

Depletion of NK cells was performed by i.p. inoculation of 200 μg anti-NK1.1 mAb PK136 or 20 μl anti-asialo GM1 antisera (Wako), as indicated. Antibody treatment was done 1 d before or on the indicated days after virus infection. For depletion of T cells, mice were injected i.p. with 200 μg anti-CD4 mAb GK1.5 and 200 μg anti-CD8 mAb 2.43 1 d before infection. For NKG2D blockade mice were inoculated with purified 200 μg CX5 Ab 1 d before and 2 d PI.

### Isolation of liver lymphocytes.

Mice were exsanguinated from the orbital cavity to decrease the amount of blood in the liver. The liver was removed and passed through a cell strainer (BD Falcon) to obtain a single cell suspension. The cells were resuspended in 35% Percoll solution (in PBS) containing 100 U/ml heparin and centrifuged at 830 × *g* for 15 min at room temperature. The upper liquid phase was removed from the tube, the lymphocyte pellet resuspended in 0.84% NH_4_Cl solution to lyse the red blood cells, and then washed twice with medium.

### BrdU incorporation assay.

At the indicated time post-infection (PI), mice were injected with 2 mg BrdU i.p. 3 h later, spleens and lymph nodes (LNs) were removed and made into single cell suspensions. The liver lymphocytes were obtained as described above. The cells were then stained for cell surface molecules, fixed, and permeabilized using the Cytofix/Cytoperm kit (BD Pharmingen) according to the manufacturer's instructions, incubated with DNase at 37 °C for 1 h, and subsequently stained with FITC-conjugated anti-BrdU mAb (eBiosciences).

### Flow cytometry.

Determination of cytokine production by intracellular staining was done as described previously [8]. To determinate NK cell responses in LNs, intact organs were incubated at 37 °C for 1 h in media containing 10 μg/ml brefeldin A, made into single cell suspensions, stained, and analyzed as described above. To evaluate NK cell responses in the spleen, the organs were made into single-cell suspensions, RBC were lysed with 0.84% NH_4_Cl, and the lymphocytes were washed and incubated at 37 °C for 1 h with brefeldin A, followed by staining and analysis as described. To determine expression of NKG2D ligands, MEFs were infected with 0.5 pfu ECTV 189898-p7.5-EGFP for 18 h. Infected cells (∼15%) were identified by EGFP expression. Gated infected and uninfected cells were analyzed for expression of NKG2D ligands by staining with: a PE-labeled rat anti-mouse Rae-1 (IgG2a isotype, R&D Systems) or a PE-labeled rat isotype-matched control IgG2a; with rat anti-mouse MULT1 (IgG2a isotype, R&D Systems) and secondary PE-labeled donkey anti-rat IgG2a or secondary Ab alone as a control; or with mouse NKG2D-human Fc fusion protein (R&D Systems) and an APC-labeled anti-human IgG (BD) secondary Ab or secondary Ab alone as control.

### ^51^Cr release assays.

NK cells were purified from spleens using anti-CD49b-conjugated microbeads and a LS column (Miltenyi Biotec) according to the manufacturer's instructions and were stained for flow cytometric analysis with PE-conjugated anti-NKG2D mAb CX5. NK cells were resuspended in RPMI 10, and serially diluted in round-bottom 96-well plates in triplicate in 100 μl/well. The indicated target cells were prepared by incubation with 200 μl ^51^Cr (0.1 mCi) in 100 μl of FCS for 2 h. Cells were thoroughly washed, resuspended in RPMI 10, and 50 μl (5×10^3^ targets) were added to the wells containing effector cells. The plates were incubated at 37 °C for 4 h. Where indicated, 20 μg/ml of a neutralizing anti-NKG2D mAb (clone 191004, R&D Systems) was added at the initiation of cytotoxicity assays. 50 μl supernatants were transferred to white 96-well plates containing 75-μl Microscint-40 scintillation fluid (PerkinElmer). Controls included wells with target cells alone for spontaneous release and wells with target cells and 1% Triton-X for maximal release. Radioactivity was measured by using a Packard Topcount instrument (PerkinElmer). Specific lysis was determined by using the formula [(experimental release − spontaneous release)/(full release − spontaneous release)]×100. Three animals were used per experimental group.

### Quantitative real-time PCR (qRT-PCR).

Primers and probes for Rae-1 were purchased from Applied Biosystems. The amplicon was 95 bp and the sequence of the probe was GGAAAAGCCAAGATCAACCTCAAGG. The primers and probe for β-actin were synthesized at the DNA Synthesis Facility in the Fox Chase Cancer Center. The primers and probe used for β-actin were: sense, 5′-CACCGAGGCCCCCCT-3′; anti-sense, 5′-CAGCCTGGATGGCTACGTACA-3′, and the probe was 5′-6-FAM-AACCCTAAGGCCAACCGTGAAAAGATGA-BHQ1–3′. Total RNA extracted from infected cell lines and LNs of infected mice was treated with Dnase I, and the first-strand cDNA was synthesized by using random primers. qRT-PCR was carried out by using the ABI 7500 (Applied Biosystems). The cycling conditions for real-time PCR were: 50 °C for 10 min, followed by 45 cycles of 95 °C for 30 s, and 60 °C for 2 min. Data were analyzed by using the Sequence Detection v1.2 Analysis Software (Applied Biosystems).

### Statistical analysis.

We used a two-tailed *t* test for two samples for means with a confidence level (alpha) of 0.05 using Excel Analysis Tool Pack (Microsoft). Differences in survival and disease were determined at the FCCC Biostatistics and Bioinformatics Facility using Log Rank Test with the STATE SE/9.2 software. In all cases, differences were considered significant when *p-*values were ≤ 0.05.
